# Steroid responsive encephalopathy associated with autoimmune thyroiditis following ipilimumab therapy: a case report

**DOI:** 10.1186/s13104-015-1283-9

**Published:** 2015-07-26

**Authors:** David Carl, Carsten Grüllich, Steffen Hering, Martin Schabet

**Affiliations:** Department of Neurology, Klinikum Ludwigsburg, Posilipostraße 4, Ludwigsburg, Germany; National Cent Tumor Diseases (NCT), Heidelberg, Germany; Department of Internal Medicine, Klinikum Bietigheim, Bietigheim-Bissingen, Germany

**Keywords:** Ipililumab, Steroid responsive encephalopathy associated with autoimmune thyroiditis, SREAT, Hypophysitis, Hashimoto’s encephalopathy, Hashimoto’s thyroiditis

## Abstract

**Background:**

Ipilimumab is a cytotoxic T-lymphocyte-associated protein 4 receptor antibody used for immunotherapy in cancer. Several immune-related adverse events are known. Steroid responsive encephalopathy associated with autoimmune thyroiditis is an autoimmune encephalopathy associated with Hashimoto’s Disease and elevated serum levels of the related antibodies (anti-thyroid-peroxidase antibody or anti-thyroglobulin antibody). Our case implies that steroid responsive encephalopathy associated with autoimmune thyroiditis may be another previously unreported side effect of ipilimumab therapy.

**Case presentation:**

We report the case of a 64 years old caucasian patient with prostatic cancer who received ipilimumab therapy in a clinical trial. He presented with aphasia, tremor and ataxia, myocloni, hallucinations, anxiety and agitation in turns with somnolence. Cranial nerves, deep tendon reflexes, motor and sensory functions were normal. Electroencephalography showed background slowing but no epileptic discharges. Brain magnetic resonance imaging was normal and showed no signs of hypophysitis. Cerebrospinal fluid findings ruled out infection and neoplastic meningitis. Anti-thyroid antibodies (anti-thyroid-peroxidase antibody and anti-thyroglobulin antibody) were heavily increased. Assuming steroid responsive encephalopathy associated with autoimmune thyroiditis the patient was treated with 1,000 mg methylprednisolone i.v. for 3 days and continued with 1 mg/kg orally. On the 3rd day of treatment the patient’s condition started to improve. Within the next few days he gradually returned to his previous state, and electroencephalography eventually showed only slight slowing. Seven months later the patient’s condition was stable, and anti-thyroid antibodies were no more detectable.

**Conclusion:**

Steroid responsive encephalopathy associated with autoimmune thyroiditis may be a hitherto unrecognized complication of ipililumab treatment and should be taken into consideration in patients developing central nervous symptoms undergoing this treatment.

## Background

Steroid responsive encephalopathy associated with autoimmune thyroiditis (SREAT) was previously referred to as Hashimoto’s encephalopathy, and is a rare disease. It is defined by the occurrence of anti-thyroid antibodies [anti-thyroid-peroxidase antibody (anti-TPO), anti-thyroglobulin antibody (anti-TG), anti-α-enolase antibodies], and the response to steroids. Symptoms and signs include cognitive and memory dysfunction, confusion, psychiatric disturbances, focal and generalized epileptic seizures, stroke-like episodes, myoclonus, ataxia, tremor, and choreatiform movements. SREAT may present as an acute, subacute or even chronic illness. The most consistent cerebrospinal fluid (CSF) finding is an elevated protein level without pleocytosis. The immunoglobulin G index is usually normal, oligoclonal bands are found occasionally. Magnetic resonance (MRI) and computed tomography (CT) imaging of the brain show the whole range of findings from entirely normal to various nonspecific abnormalities including cerebral atrophy, white matter abnormalities both focal and confluent, cortical irregularities, and vasculitic changes [1, 2].

The association of the disease with anti-thyroid antibodies and the responsiveness of SREAT to steroids or other therapies such as plasmapheresis supports the hypothesis that this is a disorder that involves immunopathogenic mechanisms. However, it is still debated whether anti-thyroid antibodies represent an immunological epiphenomenon in a subset of patients with encephalopathic processes or whether they are really associated with pathogenic mechanisms of the disorder [1, 2].

Ipilimumab (MDX-010, “Yervoy”; Bristol-Myers Squibb, Princeton, New Jersey, USA) is licensed by US Food and Drug Administration and European Medicines Agency for the treatment of metastatic melanoma. It is a fully human IgG_1_ monoclonal antibody against the cytotoxic T-lymphocyte-associated protein 4 (CTLA-4) receptor. CTLA-4 blockage results in enhanced activation and expansion of T-lymphocytes. Adverse effects of ipilimumab are associated with the enhanced immune response. Mainly lymphocyte related inflammations arise, e.g. dermatitis, enterocolitis, hepatitis, hypophysitis and others. Adverse effects can be managed with steroid therapy [3, 4]. Our case may present a previously not reported side effect of ipilimumab.

## Case presentation

Our 64 year old caucasian patient had a history of prostate cancer, diagnosed in 2001 [cT3, cN2, M0, G3, Gleason 9; prostate-specific antigen (PSA) 151 µg/l]. He was successfully treated with local radiotherapy combined with chemical castration for 1 year (complete remission, PSA nadir 0.01 µg/l). In 2006 a biochemical relapse occurred that was treated by androgen ablation. In 2011 Cholin-Pet CT showed local relapse as well as suspect lymph nodes. PSA was increased to 28.9 µg/l. The patient was enrolled into a clinical trial of ipilimumab for asymptomatic castration refractory prostate cancer (CA184095; NCT01057810) at the National Center for Tumor Diseases (NCT), Heidelberg. Starting in August 2011 he received four infusions of ipilimumab (10 mg/kg) at 3-week-intervals followed by three infusions at 3-month-intervals. Ipilimumab treatment was effective as PSA dropped and CT showed tumor regression, but the patient also experienced severe adverse effects: he had epigastric pain and pruritus, followed by heavy diarrhea, one episode leading to exsiccosis and prerenal failure. In November 2011 hypophysitis was diagnosed by decreased thyroid-stimulating hormone (TSH) levels and low thyroxine levels, an abnormal thyrotropin-releasing hormone test, and low cortisone blood levels. Thyroid volume was normal (24 ml) with presence of a small nodule (0.5 ml). Anti-thyroid antibodies (anti-TPO and anti-TG) were elevated (Table 1). The patient was started on l-thyroxine at 50 µg/days and gradually increased to 100 µg once a day. He also received Prednisolone 50 mg once daily, which was gradually reduced and switched to hydrocortisone 20 mg/days in January 2012. Following a normal Metopirone test in June 2012, hydrocortisone was tapered and stopped in July 2012. Therapy with l-thyroxine 100 µg/days was continued. The study medication was continued within the maintenance phase until July 2012.

**Table 1:** Laboratory findings

Serum parameter	April 2011	November 2011	June 2012	November 2012	June 2013
TSH (mU/l)	1.31 (0.3–4.2)	*0.01* (0.4–4.0)	*0.05* (0.47–4.7)	*0.23* (0.47–4.7)	*0.027* (0.47–4.7)
fT3 (ng/l)	3.08 (2.45–5.93)	2.75 (2.0–4.2)	–	2.91 (2.45–5.93)	*6.05* (2.45–5.93)
fT4 (ng/l)	11.1 (7.8–24.4)	10.57 (8–18)	15.6 (7.8–24.4)	18.2 (7.8–24.4)	11.57 (7.8–24.4)
Anti-TG (IU/ml)	–	*285* (<60)	–	*1,902* (<115)	–
Anti-TPO (IU/ml)	–	*410* (<60)	–	*138* (<34)	27 (<34)
TRAbs (IU/l)	–	<0.3 (<1.75)	–	<0.3 (<1.75)	<0.3 (<1.75)
PSA (µg/l)	*13.4* (<4)	0.11 (<4)	–	0.004 (<4)	<0.002 (<4)
Testosteron (ng/ml)	–	*0.11* (2–7)	–	*<0.1* (3–10.6)	–
Prolaktin	–	179 (43–375) mU/l	–	11.20 (3–25) ng/ml	–
Cortisol (ng/ml)	–	*12.1* (50–250)	–	–	*0.5* (50–250)

Ranges of normal values are given in brackets. Abnormal values are printed in italics.

*fT3* free triiodothyronine, *fT4* free thyroxine, *TRAbs* TSH receptor antibodies.

–: not determined.

In August 2012, following surgery of an epigastric hernia the patient had severe postoperative intraabdominal bleeding leading to hypovolemic shock followed by sepsis. He physically recovered within 3 weeks, but slight personality changes remained. He slowly deteriorated suffering from adynamia, memory disturbances, and fluctuating disorientation. When the patient was admitted to our hospital in November 2012 he was bedridden, somnolent and disoriented. He had episodes of agitation and hallucinations, and showed myocloni and intermittent focal seizures of his right arm. Deep tendon reflexes were normal, but Babinski’s sign was intermittently positive. He had no paresis. Cranial CT scan was normal. Electroencephalography (EEG) showed generalized slowing with prevailing of slow theta and delta waves. CSF cell count, glucose, and lactate were normal; protein was 85.2 mg/dl (normal <50). Serum C-reactive protein was 76 mg/l (normal <5), erythrocyte sedimentation rate 86 mm/h. Leukocyte count was 10.2/µl, erythrocyte count 4.08/ng, haemoglobin 11.4 g/dl. Renal and liver functions were normal. Anti-thyroid antibodies (anti-TPO and anti-TG) in serum were markedly elevated (Table 1). Antibodies associated with vasculitis proved negative. Anti-NMDA-, VGKC- or onconeural-antibodies were neither determined in serum nor in CSF. Cranial MRI scan showed mild microangiopathic changes, an old lacunar infarction in the right thalamus, and a normal pituitary gland. CT-Scan of the abdomen and thorax showed stable disease at the primary site, and no metastasis. PSA was 0.004 µg/l.

Levetiracetam 1,000 mg bid stopped focal seizures, but myoclonus and all other symptoms and signs remained. The clinical and laboratory settings lead us to assume SREAT. On the 3rd day after admission we started treatment with 1,000 mg methylprednisolone intravenously for 3 days. On the 6th day we continued with Prednisolon 100 mg orally and tapered to 60 mg once a day within a week. Episodes of hallucinations and anxious agitation were treated with haloperidol 2 mg and lorazepam 0.5 mg tid. On the 3rd day of steroid treatment the patient began to improve. On the 5th day he was able to communicate coherently. The psychiatric symptoms disappeared, and the respective medication was discontinued. The patient was still temporally disorientated and had slowed psychomotor functions, but gradually improved. The EEG improved. Fifteen days after the start of steroids the patient was discharged. Prednisolone was tapered by 10 mg every 4 weeks.

The patient’s condition further improved. At follow-up in June 2013 he only had subtle memory deficits and was slightly temporally disoriented but was fully aware of this and capable of coping in his daily living. The anti-TPO concentration was back to normal (Table 1). Ipilimumab therapy was not resumed and the patient received no other treatment for his prostate cancer after these complications. He remained in complete remission with normal PSA values until the last follow-up so far in July 2014.

## Conclusions

Clinical symptoms and signs, typically elevated anti-thyroid antibodies and the exclusion of infectious encephalitis, meningeal carcinomatosis and ischemia lead us to diagnose SREAT in our patient. The prompt and continuous improvement of symptoms and signs with steroid treatment, also favored the diagnosis of SREAT. As we did not test for onconeural antibodies we cannot rule out paraneoplastic encephalitis which has also been reported in patients with prostate cancer [5]. However, paraneoplastic syndromes are rare in this cancer. Classical syndromes include cerebellar degeneration, brainstem syndromes and peripheral neuropathy, in descending order of frequency. In addition, steroid therapy alone is usually not effective and prognosis is poor in classical paraneoplastic encephalitis [5, 6].

Prior to ipilimumab therapy our patient had normal thyroid hormone levels (Table 1). Anti-TPO and anti-TG antibodies were not determined at this point. On diagnosis of hypophysitis and later on SREAT both TSH and thyroid hormone levels were expectably low, and anti-TPO and anti-TG antibodies were elevated (Table 1). Though up to 7% of the normal population [7] carry these antibodies without having autoimmune thyroiditis ipilimumab therapy in our patient could have boosted these antibodies and triggered both hypophysitis and thyroiditis [3, 4].

Ipilimumab therapy has been associated with two cases of Hashimoto’s thyroiditis and several cases of Graves’ disease caused by anti-TSH receptor antibodies [8, 9].

Encephalitis has been seen in a patient following two doses of ipilimumab [10]. He “developed confusion and lethargy without abnormalities on central nervous system imaging, or in the cerebrospinal fluid”. Steroid therapy resolved symptoms, so it could have well been SREAT. The authors did not refer to thyroid hormones and anti-thyroid antibodies. Recently, immune-related meningitis and Guillian–Barré syndrome have been reported in patients treated with ipilimumab [11]. Therefore, neurological complications of ipilimumab therapy may be more frequent than realized so far.

According to its approval report ipilimumab should be avoided in patients with active autoimmune disease and used with caution in patients with a history of autoimmune disease. The manufacturer states that ipilimumab was tested in patients with adequately controlled hypothyroidism and “seemed to be unproblematic” [12]. Testing anti-thyroid antibodies before starting treatment and on follow-up may improve its safety. This would help to recognize autoimmune thyroiditis and SREAT as possible rare but relevant treatment-related complications.

## Consent

Written informed consent was obtained from the patient for publication of this Case Report and any accompanying images. A copy of the written consent is available for review by the Editor-in-Chief of this journal.

## Abbreviations

SREAT: steroid responsive encephalopathy associated with autoimmune thyroiditis
anti-TPO: anti-thyroid-peroxidase antibody
anti-TG: anti-thyroglobulin antibody
CSF: cerebrospinal fluid
CTLA-4: cytotoxic T-lymphocyte-associated protein 4
EEG: electroencephalography
MRI: magnetic resonance imaging
CT: computed tomography
PSA: prostate-specific antigen
TSH: thyroid-stimulating hormone
